# Manifestations of Laser-Induced Graphene under Ultraviolet Irradiation of Polyimide with Varied Optical Fluence

**DOI:** 10.3390/nano12081241

**Published:** 2022-04-07

**Authors:** Ilija R. Hristovski, Luke A. Herman, Michael E. Mitchell, Nikolai I. Lesack, Jason Reich, Jonathan F. Holzman

**Affiliations:** Integrated Optics Laboratory, School of Engineering, University of British Columbia, Kelowna, BC V1V 1V7, Canada; ilijahri@me.com (I.R.H.); lukeherman1997@gmail.com (L.A.H.); mikemitch@outlook.com (M.E.M.); nlesack123@hotmail.com (N.I.L.); jasonreich@outlook.com (J.R.)

**Keywords:** graphene, laser-induced graphene, Raman spectroscopy, optical fluence

## Abstract

In this work, we put forward a rigorous study on ultraviolet (355-nm) laser irradiation of polyimide for the realization of high-quality laser-induced graphene (LIG) with micron-scale features. High-quality material and micron-scale features are desirable—but often at odds—given that small features demand tightly focused beam spots, with a predisposition to ablation. As such, we investigate the synthesis of LIG by correlating the material characteristics, as gleaned from scanning electron microscopy and Raman spectroscopy, to the incident optical fluence, as a measure of applied optical energy per unit area. The study reveals that high-quality LIG, with ratios of Raman 2D-to-G peak heights approaching 0.7, can be synthesized with micron-scale features, down to 18 ± 2 μm, given suitable attention to the optical fluence. Optimal characteristics are seen at optical fluences between 40 and 50 J/cm^2^, which promote graphenization and minimize ablation. It is hoped that these findings will lay a foundation for the application of LIG in future integrated technologies.

## 1. Introduction

Graphene is a two-dimensional allotrope of carbon with electrical and optical properties that are extraordinary [1,2,3]. It has gained particular attention in optoelectronics due to its unique band structure, with a bandgap energy of zero. Such a structure yields minimal wavelength dependence in its absorption processes [4] and ultrafast charge carrier dynamics in its relaxation processes, given the predominance of energy and momentum relaxation [4]. This has led to numerous studies on the synthesis of graphene, via electrochemical exfoliation of graphite [5] and electrodeposition [6], the modification of graphene, by way of covalent chemistry [7] and photochemical modification [8], and the application of graphene, in photodetectors [9] and optical modulators [10].

Of the various means to synthesize graphene, those applying laser writing are particularly advantageous. They enable direct patterning of graphene with the potential for micron-scale features. The concept was introduced by Lin et al. in forming laser-induced graphene (LIG) within a polyimide film by way of a CO_2_ laser, at a wavelength of 10.6 µm [11]. These findings have since led to many attempts at reducing the size of the beam spot and, thus, the LIG feature size by moving from the long infrared wavelength to the shorter ultraviolet wavelengths of 355 nm [12] and 404 nm [13]. The latter study used stationary irradiation for a set duration of time to apply a finite optical fluence (i.e., energy per unit area) and in doing so realized LIG spots with sizes down to 12 µm. Nevertheless, the extrapolation of such work to the direct writing of LIG lines is nontrivial. This is because the translation velocity used to write such lines defines the level of graphenization, which requires a sufficiently high optical fluence, and ablation, wherein low velocities are predisposed to photothermal ablation (at a relatively high threshold optical fluence) and high velocities are predisposed to photochemical-induced ablation (at a relatively low threshold optical fluence) [14]. Ultimately, the direct writing of LIG lines is subject to a complex interplay between graphenization and ablation—with the pursuits of micron-scale features and high-quality graphene material often at odds.

The (desired) LIG generation can be brought about via photothermal effects and/or photochemical effects. Photothermal effects play a dominant role under visible or infrared irradiation, at wavelengths above 400 nm, as the photon energies are less than the dissociation energies of chemicals bonds in the material [12,15,16]. The deposited energy from the laser pulses, i.e., the optical fluence, then excites vibrational modes and raises the temperature. This can yield a transition to an intermediate amorphous carbon state, under certain circumstances [17,18], and the desired transition from sp^3^ to sp^2^ bonds in the crystalline graphene state [11]. In contrast, photothermal and photochemical effects can play a role under ultraviolet irradiation, at wavelengths below 400 nm, given that the photon energies are often greater than the dissociation energies of the constituent chemical bonds [12,15,16]. Although less is known about LIG generation under ultraviolet irradiation [19], the physical features suggest a dominance of photothermal effects at low optical fluences and contributions from photochemical effects at high optical fluences—mediated by the ionization of nitrogen in the air and subsequent reactions [12].

The (undesired) ablation can also occur during LIG generation and arise from photothermal and/or photochemical effects. Shin et al. showed that photothermal and photochemical effects contribute to the ablation of polyimide at a wavelength of 355 nm given low and high optical fluences, respectively [20]. Zhang et al. then used the metric of linear laser energy density (LLED), as the ratio of laser beam power to translation velocity, to attribute ablation to photochemical or photothermal effects [14]. For the ablation of polyimide under 355 nm irradiation, they concluded that photothermal effects dominate at LLED values greater than 6 J/m, photochemical effects dominate at LLED values less than 3 J/m, and both effects arise at LLED values between these limits.

In this work, we rigorously characterize the synthesis of LIG in polyimide under ultraviolet irradiation as a function of the incident optical fluence (i.e., applied optical energy per unit area). This is done to balance the (desired) graphenization [21] and (undesired) ablation [18] and, thus, realize high-quality LIG with micron-scale features.

## 2. Materials and Methods

**Figure 1:**
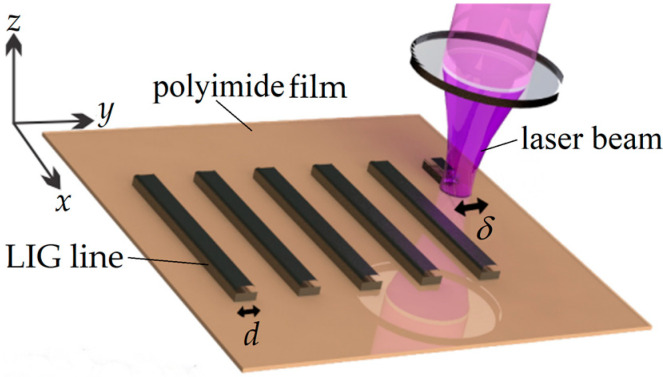
A schematic of the LIG generation system is shown. The 355 nm laser beam is focused to a fixed spot in the polyimide film, with a full-width-at-half-maximum (FWHM) of *δ*. The film is translated to create LIG lines along the *x*-direction having a width of *d* in the *y*-direction.

The LIG generation system for this work uses a pulsed ultraviolet laser (Oxford Lasers Ltd., A-355 Laser Micro-machining System, Didcot, Oxfordshire, UK) with a wavelength of 355 nm, a focal length of 100 mm, an output beam diameter of 35 mm, a pulse duration of 33 ns, and a repetition rate of 50 kHz. A 2-cm^2^ section of 75-µm-thick polyimide film (Kapton, 18-3F-24, CS Hyde Company, Lake Villa, IL, USA) is translated via a three-dimensional micro-positioning system, whereas the laser beam is focused to a spot in the film. The polyimide film is translated in the *x*-*y* plane, to vary the location of the beam spot in the film and the *z*-direction, to vary the size of the beam spot in the film. A characterization of the beam spot is given in Appendix A. The LIG generation system, illustrated in Figure 1, translates the polyimide film at a velocity of *v* = 11 mm/s (in the *x*-direction) to form LIG lines of width *d* (in the *y*-direction). During this translation, the polyimide film is irradiated by the laser beam at normal temperature and pressure (NTP), i.e., a temperature of 20 °C and pressure of 1 atm.

The intensity of the laser beam in the polyimide film is characterized by a Gaussian distribution in the *x*-*y* plane with a maximal intensity of *I*_0_ at its centre and a beam spot size of *δ* = (2ln(2))^1/2^*ω*, where *ω* is the radius at which the intensity falls to 1/e^2^ of the maximal intensity and *δ* is its full-width-at-half-maximum (FWHM). The intensity distribution can then be defined at the position (*x*,*y*) and time *t* by

(1) $$I(x,y,t)=I_{0}\cdot \;e^{-2({\left(x-vt\right)}^{2}+y^{2})/\omega^{2}}$$

where *P* = π*ω*^2^*I*_0_/2 = π*δ*^2^*I*_0_/(4ln(2)) is the total laser beam power over the cross-section of the beam spot. The intensity and power are quoted in this work as time-averaged quantities (over the train of laser pulses) incident on the polyimide surface. Given the translation of the polyimide film along the *x*-direction, we can integrate the intensity distribution in Equation (1) over all time, *t*, to define the incident optical fluence as a function of *y* via

(2) $$\begin{array}{ll} \Phi (y) & =\int_{-\infty }^{+\infty }I(x,y,t)\;dt=I_{0}e^{-2y^{2}/\omega^{2}}\int_{-\infty }^{+\infty }e^{-2{\left(x-vt\right)}^{2}/\omega^{2}}\;dt=I_{0}e^{-2y^{2}/\omega^{2}}\frac{1}{v}\int_{-\infty }^{+\infty }e^{-2t\prime^{\;2}/\omega^{2}}\;dt\prime \\ & =I_{0}e^{-2y^{2}/\omega^{2}}\frac{1}{2v}\sqrt{\frac{\pi }{2}}\omega {\left[\mathrm{erf}\left(\frac{\sqrt{2}t\prime }{\omega }\right)\right]|}_{-\infty }^{+\infty }=\;I_{0}\sqrt{\frac{\pi }{2}}\cdot \frac{\omega }{v}\cdot e^{-2y^{2}/\omega^{2}}=\Phi_{0}\cdot e^{-2y^{2}/\omega^{2}}, \end{array}$$

where the maximal optical fluence (at *y* = 0) is

(3) $$\Phi_{0}=\sqrt{\frac{4\ln(2)}{\pi }}\;\cdot \;\frac{P}{v\delta }\approx 0.9394373\;\cdot \;\frac{P}{v\delta }$$

The optical fluence in Equation (3) is a time-averaged quantity, similar to the intensity and power. Moreover, its finite optical energy per unit area arises from the translation velocity of the polyimide film and not the laser pulse duration, as seen in studies with fixed numbers of laser pulses. Thus, our definition of optical fluence parallels the *dynamic fluence* used by Tiliakos et al. for 10.6-μm LIG generation [22] and Lee et al. for 355 nm LIG generation [23]. The scaling factor of (4ln(2)/π)^1/2^ in Equation (3) arises from the Gaussian laser beam and is approximated by unity in [22] and [23]. In the remainder of this work, we characterize the LIG in terms of the optical fluence, *Φ*_0_, as it encapsulates the combined effects of laser beam power, *P*, translation velocity, *v*, and spot size, *δ*. The laser beam power is set at *P* ≈ 245 mW, and the beam spot size is varied from *δ* ≈ 88.7 down to 30.7 µm to vary the optical fluence from *Φ*_0_ ≈ 23.6 to 68.2 J/cm^2^, unless stated otherwise. The translation velocity is set at *v* = 11 mm/s, as this small value mitigates mechanical vibrations and lessens their effects on the microscopic scale of our system.

## 3. Results and Discussion

**Figure 2:**
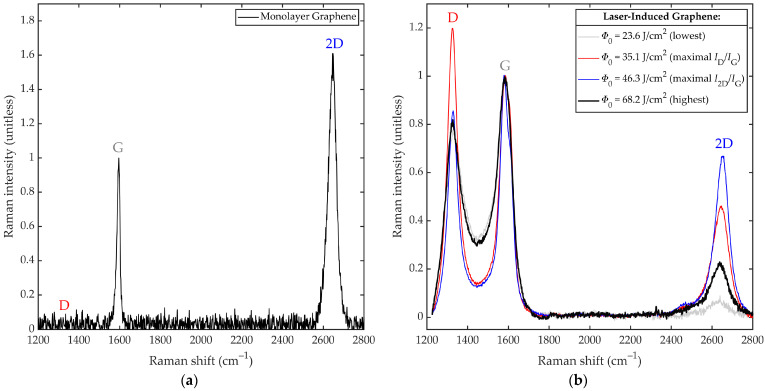
Results from Raman spectroscopy are shown as Raman intensity versus Raman shift. The results are shown for (**a**) monolayer graphene and (**b**) four LIG lines having been generated by select optical fluences of *Φ*_0_ = 23.6, 35.1, 46.3, and 68.2 J/cm^2^. The peaks appearing at approximately 1350, 1580, and 2650 cm^−1^ are designated by D, G, and 2D, respectively.

Raman spectroscopy is used to characterize the LIG quality under varied optical fluence. The results were acquired from a Raman spectrometer (Renishaw inVia Raman Microscope, Wotton-under-Edge, UK) with excitation at a power of 22.5 mW and wavelength of 632.8 nm, and focusing by an objective lens with a magnification of 20×, numerical aperture of 0.4, and spatial resolution of 1 μm^2^. This gives a spectral resolution of 2 cm^−1^. Figure 2a,b show our measurements of Raman intensity versus Raman shift for monolayer graphene (Graphenea, Monolayer Graphene on Quartz, Cambridge, MA, USA) and LIG, respectively, given LIG generation at optical fluences of *Φ*_0_ = 23.6, 35.1, 46.3, and 68.2 J/cm^2^. Figure 2a exhibits G and 2D peaks near 1580 and 2650 cm^−1^, respectively, with the emergence of these two peaks (and only these peaks) indicating the presence of near-ideal monolayer graphene. (Such results are in agreement with those quoted by the supplier, Graphenea, Cambridge, MA, USA.) Figure 2b exhibits prominent D, G, and 2D peaks near 1350, 1580, and 2650 cm^−1^, respectively, from which characteristics of LIG can be gleaned. The D peak arises in graphene near 1350 cm^−1^ due to edges and/or defects. It can manifest as a D_1_ peak from edges of monolayer graphene or superimposed D_1_ and D_2_ peaks from edges of graphite, giving a broader overall peak [24]. The ratio of D-to-G peak heights is often used to gauge the fraction of graphite and/or disordered graphene (as a measure of LIG quality). The G peak arises in graphene near 1580 cm^−1^ due to doubly degenerate *E*_2G_ phonons at the Brillouin-zone centre [25]. It can show a slight redshift due to increasing numbers of layers [26] and/or be superimposed with a subtle D’ peak at 1620 cm^−1^, from intravalley phonons and scattering in the K valley due to disorder [27]. The G peak height is often used as a benchmark of comparison for the D and 2D peak heights. The 2D peak arises in graphene near 2650 cm^−1^ as a single narrow peak [24]. It can manifest in ideal graphene, unlike the D peak, as phonons with opposing wavevectors can meet momentum conservation without defects [28]. With added layers and/or bulk graphite, the 2D peak is seen to split into 2D_1_ and 2D_2_ peaks. The overlapping 2D_1_ and 2D_2_ peaks yield an overall 2D peak that is redshifted and broadened—as an indicator of inferior LIG quality [29,30]. The ratio of 2D-to-G peak heights and the width of the 2D peak are often used to gauge the proportion of ordered graphene in LIG.

We can see from Figure 2b that the heights of the above three peaks vary with optical fluence, *Φ*_0_. The lowest optical fluence, *Φ*_0_ = 23.6 J/cm^2^, gives a low D peak and very low 2D peak, with respect to the G peak, suggesting that weak graphitization and very weak graphenization occurred. At the next optical fluence, *Φ*_0_ = 35.1 J/cm^2^, the D peak rises to a great height and the 2D peak rises to a moderate height, indicating strong graphitization and moderate graphenization, respectively. At the optical fluence of *Φ*_0_ = 46.3 J/cm^2^, the D peak falls to a moderate height, whereas the 2D peak rises to a great height, indicating moderate graphitization and strong graphenization, respectively. At the highest optical fluence, *Φ*_0_ = 68.2 J/cm^2^, the D peak is largely unchanged and the 2D peak falls, suggesting that the added optical fluence decreases the fraction of ordered graphene due to ablation.

**Figure 3:**
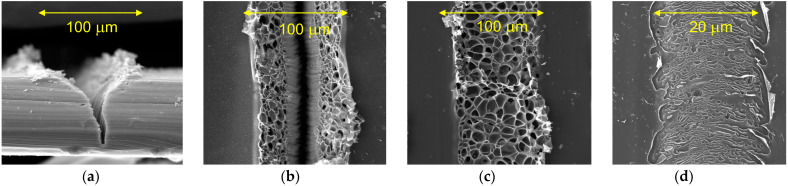
Scanning electron microscope (SEM) images of LIG lines generated by (**a**) an optical fluence that is well above the ablation threshold, at 58 J/cm^2^, yielding an ablated trench, (**b**) an optical fluence that is slightly above the ablation threshold, at 46.3 J/cm^2^, yielding a narrow ablated trench in the centre and micron-sized pores of LIG on either side, (**c**) an optical fluence that is slightly below the ablation threshold, at 44.8 J/cm^2^, yielding only micron-sized pores of LIG, and (**d**) a LIG line generated by an optical fluence that is below the ablation threshold, at 27.4 J/cm^2^, with its reduced laser beam power and spot size yielding a width of *d* ≈ 18 ± 2 μm.

The spatial characteristics of the LIG lines can be seen in the scanning electron microscope (SEM) images of Figure 3. Figure 3a shows the cross-section of a LIG line made with an optical fluence that is well above the ablation threshold (at 58 J/cm^2^). The SEM image shows a deep trench that has been ablated into the polyimide. Figure 3b shows a LIG line with a width of *d* ≈ 95 ± 5 µm generated by an optical fluence that is slightly above the ablation threshold (at 46.3 J/cm^2^). Such an optical fluence yields a narrow trench from ablation in the centre of the LIG line, where the optical fluence is highest, and graphene with micron-sized pores on either side, where the optical fluence is less. Figure 3c shows a LIG line with a width of *d* ≈ 85 ± 5 µm generated by an optical fluence that is slightly below the ablation threshold (at 44.8 J/cm^2^). Ablation is absent and micron-sized pores can be seen across its width. Figure 3d shows a LIG line generated by scaling down the laser beam power and spot size to give a lower optical fluence (at 27.4 J/cm^2^). The result is a LIG line without ablation and nanometre-sized pores across its width of *d* ≈ 18 ± 2 µm. Further details on how the widths of these LIG lines are calculated are given in Appendix B.

Overall, we find that the pore sizes in the LIG lines scale up with increasing optical fluence. This agrees with the literature, from the earliest work [11] where the nanometre-sized pores expanded from increased optical power (and thus increased optical fluence) to more recent work [23] where the micron-sized pores expanded from decreased translation velocity (and thus increased optical fluence). Such increases in pore size have been attributed to the escape of gases, including CO and H_2_, which becomes more prevalent at higher optical fluences [12,17,23]. At the same time, we find that the pore size is negatively correlated to the bonding strength of a LIG line, whereby larger pores show weaker bond strength and potentially delamination. Namely, our tests according to the ASTM D3359-17 standard, with an adhesive tape having a peel strength between 6.34 and 7.00 N/cm, gave failures for the wider LIG lines and passes for the narrower LIG lines.

**Figure 4:**
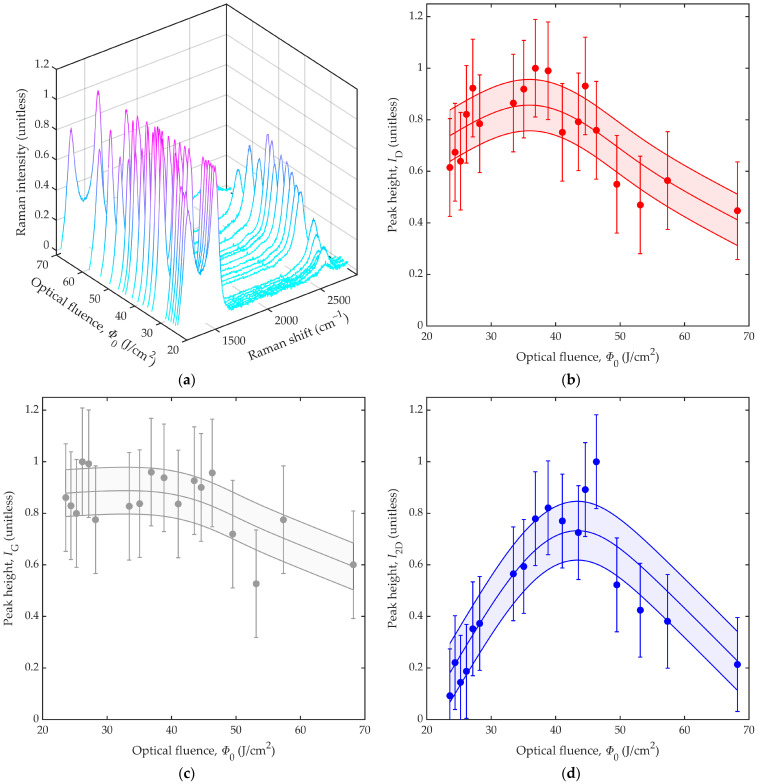
Results from Raman spectroscopy are shown for LIG lines generated with various values of optical fluence, *Φ*_0_. The results show (**a**) Raman intensity versus Raman shift and optical fluence, *Φ*_0_, (**b**) D peak height, *I*_D_, versus optical fluence, *Φ*_0_, (**c**) G peak height, *I*_G_, versus optical fluence, *Φ*_0_, and (**d**) 2D peak height, *I*_2D_, versus optical fluence, *Φ*_0_. In (**b**–**d**), the results are characterized by markers and error bars, as well as curves for the fit (middle curve), the fit minus the standard deviation (lower curve), and the fit plus the standard deviation (upper curve). Thus, the shaded regions between the lower and upper curves portray uncertainty at one standard deviation from the fit.

The spectral characteristics of the LIG lines are illustrated by the Raman peak heights shown in Figure 4. Figure 4a shows the results by way of Raman intensity as a function of Raman shift and optical fluence, *Φ*_0_. Here, we see several trends for increasing optical fluence: the D peak rises slightly over low optical fluences and then falls over high optical fluences; the G peak remains largely unchanged; and the 2D peak rises over low optical fluences and then falls slowly over high optical fluences. For added clarity, the individual trends for the D peak height, *I*_D_, G peak height, *I*_G_, and 2D peak height, *I*_2D_, in Figure 4a are presented as a function of optical fluence, *Φ*_0_, within Figure 4b–d, respectively.

The experimental results shown here are from 65 samples of LIG lines generated at 18 distinct optical fluences, with three to five samples of LIG lines used for each optical fluence. The markers denote the mean values from the samples at each optical fluence, with the lower/upper bounds on the error bars denoting the mean minus/plus the standard deviation across all 65 samples. The results used to generate the figures are shown in Appendix C as plots of Raman intensity versus Raman shift for each optical fluence. The overall trend in each figure is characterized by an empirical spline (polynomial) fit and the standard deviation of the 18 experimental datapoints with respect to this fit. The central curve denotes the fit, the lower curve denotes the fit minus the standard deviation, and the upper curve denotes the fit plus the standard deviation. Thus, the overall trends can be interpreted from the central curves, with the shaded regions between the lower and upper curves portraying uncertainty at one standard deviation from the fit. In Figure 4b, we see the D peak height, *I*_D_, rise over a low-optical-fluence regime, from *Φ*_0_ ≈ 23.6 to 40.0 J/cm^2^, indicating growth of graphite and disordered graphene, then fall over a high-optical-fluence regime, from *Φ*_0_ ≈ 40.0 to 68.2 J/cm^2^. In Figure 4c, we see the G peak height, *I*_G_, remain largely unchanged over the low-optical-fluence regime and then fall slightly over the high-optical-fluence regime. In Figure 4d, we see the 2D peak height, *I*_2D_, rise over the low-optical-fluence regime and then fall over the high-optical-fluence regime. We attribute the nonmonotonic trend in 2D peak height to the promotion of graphenization in the low-optical-fluence regime and the promotion of disorder and ablation in the high-optical-fluence regime.

**Figure 5:**
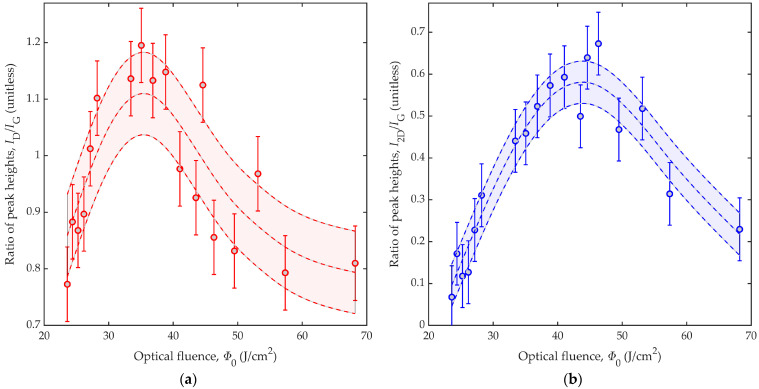
Results from Raman spectroscopy are shown for LIG lines generated with varied optical fluence, *Φ*_0_. The results show ratios of peak heights versus optical fluence, *Φ*_0_, for the (**a**) D-to-G peak height ratio, *I*_D_/*I*_G_, and (**b**) 2D-to-G peak height ratio, *I*_2D_/*I*_G_. The results are characterized by markers and error bars, as well as curves for the fit (middle curve), the fit minus the standard deviation (lower curve), and the fit plus the standard deviation (upper curve). Thus, the shaded regions between the lower and upper curves portray uncertainty at one standard deviation from the fit.

Figure 5 shows characteristics of the LIG material via ratios of Raman peak heights. We note here that the D-to-G peak height, *I*_D_/*I*_G_, is correlated to the proportion of graphite and disordered graphene, whereby reduced crystallite sizes increase the disorder and defects [31], whereas the 2D-to-G peak height, *I*_2D_/*I*_G_, is correlated to the proportion of ordered graphene, whereby *I*_2D_/*I*_G_ values greater than 1.3 are seen from monolayer graphene [31]. For example, the peak heights from our monolayer graphene in Figure 2a give *I*_2D_/*I*_G_ ≈ 1.6. Figure 5a,b show ratios of D-to-G peak height, *I*_D_/*I*_G_, and 2D-to-G peak height, *I*_2D_/*I*_G_, respectively, as a function of optical fluence, *Φ*_0_. Here, experimental results are displayed as markers with error bars and empirical spline (polynomial) fits, with the error bars and fitted curves defined in the manner described for Figure 4. The smaller error bars in Figure 5, compared with Figure 4, show the advantage of characterizing LIG material by ratios of peak heights. Namely, arbitrary scaling factors that inevitably exist between differing Raman spectra cancel in dividing the D and 2D peak heights by the G peak height. The *I*_D_/*I*_G_ results in Figure 5a suggest that the proportion of graphite and disordered graphene rise sharply across the low-optical-fluence regime, reaching a maximum near *Φ*_0_ ≈ 35 J/cm^2^, and then fall slowly across the high-optical-fluence regime. The *I*_2D_/*I*_G_ results in Figure 5b suggest that the proportion of ordered graphene rises slowly across the low-optical-fluence regime, reaching a maximum near *Φ*_0_ ≈ 44 J/cm^2^, and then falls slowly across the high-optical-fluence regime. For the LIG lines of Figure 3b–d, generated by optical fluences slightly above, slightly below, and below the ablation threshold, respectively, with LIG line widths of *d* ≈ 95 ± 5 μm, 85 ± 5 μm, and 18 ± 2 μm, respectively, we observe *I*_2D_/*I*_G_ ratios of 0.45, 0.51, and 0.29, respectively. Thus, all three LIG lines have sizable fractions of graphene, but the fraction is not entirely independent of the beam spot size. The reduction in laser beam power and spot size for the last sample, in targeting a smaller width, led to a reduction in the fraction of graphene.

Overall, the trends seen in this study agree with recent reports of LIG generation via 355 nm irradiation, although the highest-quality graphene is formed here at higher optical fluences than several studies. As examples, Carvalho et al. used a laser power of *P* = 300 mW, a translation velocity of *v* = 60 mm/s, and a spot size of *δ* = 50 µm, to generate LIG at an optical fluence of *Φ*_0_ = 9.4 J/cm^2^ [32], and Wang et al. generated LIG at a translation velocity of *v* ≈ 300 mm/s and a spot size of *δ* ≈ 40 µm, with optical fluences of *Φ*_0_ = 4.3 to 12 J/cm^2^ [12]. Indeed, the proposed work is more in line with a recent study by Lee et al. on LIG generation with optical fluences ranging from *Φ*_0_ = 18.57 J/cm^2^ to 54.16 J/cm^2^ [23]. Their work’s Supplementary Material is a particularly useful resource in linking the experimental parameters and optical fluences. As a final point, we note that the quality of LIG was characterized in this work by Raman spectra. However, it is also possible to characterize quality by electrical conductivity. In doing so, we find that the optimal optical fluences for the Raman spectra also yield the highest electrical conductivities, with values in the range of (1.5 ± 0.3) 1/(Ω·cm).

## 4. Conclusions

In this work, we put forward a study on the use of ultraviolet (355-nm) laser irradiation of polyimide for the realization of high-quality LIG with micron-scale features. The results showed that high-quality LIG can be generated using the highest allowable optical fluence, while remaining below the optical fluences that result in strong ablation. Optical fluences between *Φ*_0_ ≈ 40 to 50 J/cm^2^ meet these conditions. Such LIG can be generated with micron-scale features by reducing the beam spot size, *δ*, but this must be done with careful consideration on the power, *P*, and translation velocity, *v*. Namely, it is necessary to keep the overall optical fluence, *Φ*_0_ = (4ln(2)/π)^1/2^·*P*/(*vδ*) ≈ 0.939437·*P*/(*vδ*), below the ablation threshold. We used this principle and demonstrated LIG lines with widths down to *d* ≈ 18 ± 2 μm. However, it is worth noting that it can be difficult to control the power and translation velocity to the extent needed to prevent ablation at these small widths. Future studies may wish to leverage our results while investigating LIG generation in polyimide with controlled cooling and/or thinner films. Such control may help localize the thermal energy and realize high-quality LIG with even smaller feature sizes. Future studies may also wish to explore the role of the laser pulse duration. As mentioned at the outset of this work, LIG generation under ultraviolet illumination can be subject to photothermal effects, which are roughly independent of the laser pulse duration, and/or photochemical effects. The latter effects include (rapid) ionization and reactions of nitrogen in the air, which will depend on the peak laser beam power and thus may vary with pulse duration.

## Figures and Tables

**Figure A1:**
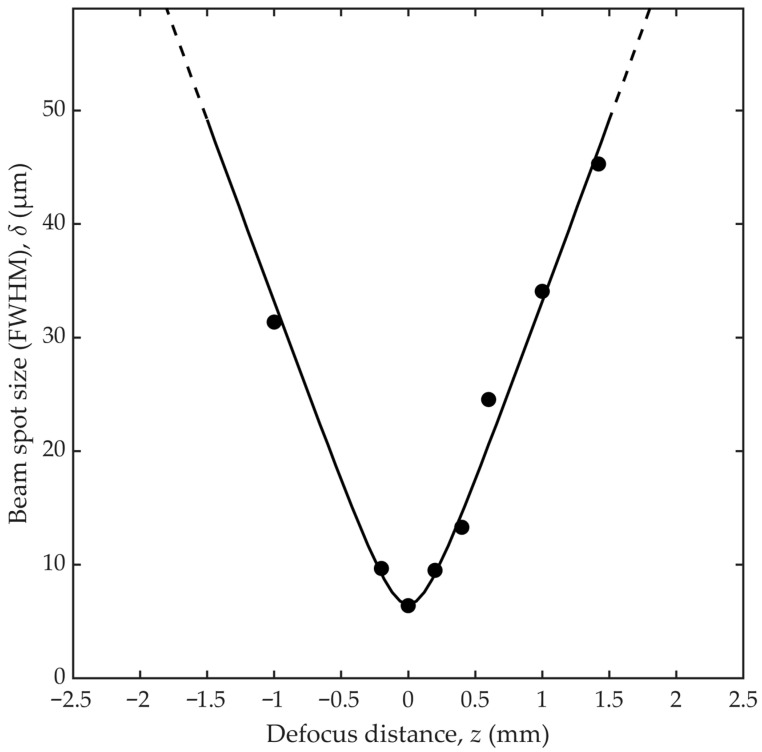
Beam spot size (FWHM), *δ*, versus defocus distance, *z*. Experimental data is displayed as black markers. The fitted equation of a focused Gaussian beam is displayed as a solid black line.

**Figure A2:**
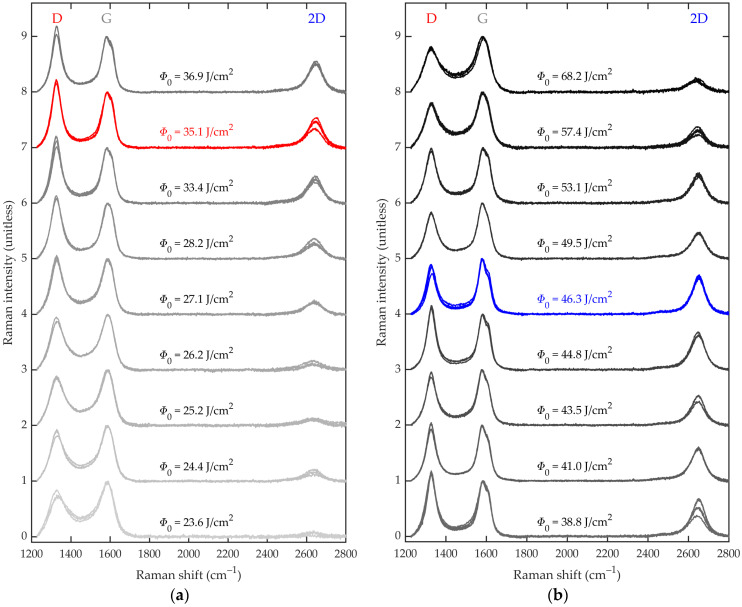
Results for Raman intensity as a function of Raman shift for LIG lines generated at optical fluences ranging from (**a**) 23.6 to 36.9 J/cm^2^ and (**b**) 38.8 to 68.2 J/cm^2^. The curves yielding maximal ratios for D-to-G peak height, *I*_D_/*I*_G_, and 2D-to-G peak height, *I*_2D_/*I*_G_, are shown in red and blue, respectively. Spectra from 65 samples are shown, with three to five spectra for each optical fluence.

## Data Availability

The data presented in this study are available on request from the corresponding author.

## Appendix A

Knife-edge scans were used to characterize the beam spot sizes. This was done with measurements of transmitted laser power as a function of the position of a knife-edge translated across the transverse plane of the beam spot. The transmitted laser power versus knife-edge position across the beam was then used to estimate the Gaussian intensity profile of the beam spot. The Gaussian intensity profile defined the radius at which its intensity fell to 1/e^2^ of the maximal intensity, *ω*, and the full-width-at-half-maximum (FWHM), *δ* = (2ln(2))^1/2^*ω*. These measurements of beam spot size were made for various values of defocus distance, *z*, as the distance away from the focus along the optical axis. We used this characterization, shown in Figure A1, to define values for the beam spot size, *δ*, and we used these *δ* to calculate the optical fluence via *Φ*_0_ = (4ln(2)/π)^1/2^·*P*/(*vδ*), where *P* is the laser power and *v* is the translation velocity.

## Appendix B

The widths of the LIG lines in the SEM images of Figure 3b–d, at *d* ≈ 95 ± 5 µm, 85 ± 5 µm, and 18 ± 2 µm, respectively, were defined by the methods shown here. The first two LIG lines had their widths quantified as mean widths from many measurements made at various positions along the lines. The widths were defined by the distance between outermost features of the line. Such measurements were relatively straightforward given their reasonably well-defined edges. In contrast, the third LIG line had poorly resolved edges, and so its width was quantified in MATLAB by the greyscale intensity from its SEM image. The greyscale intensity was distributed along the *x*-direction of Figure 1 (parallel to the LIG line) and *y*-direction of Figure 1 (perpendicular to the LIG line). A median was calculated from all the intensities along the *x*-direction at each *y* position. This gave a distribution of median intensities as a function of the *y* position (perpendicular to the LIG line). The width of the LIG line was then quantified as the FWHM of this distribution.

## Appendix C

The Raman spectra used to define peak heights and ratios of peak heights in Figure 4 and Figure 5 are shown in this appendix. Figure A2a,b show Raman intensity as a function of Raman shift for LIG lines generated at optical fluences ranging from 23.6 to 36.9 J/cm^2^ and 38.8 to 68.2 J/cm^2^, respectively. The curves yielding maximal ratios of D-to-G peak height, *I*_D_/*I*_G_, and 2D-to-G peak height, *I*_2D_/*I*_G_, are shown in red and blue, respectively. The spectra from 65 samples are shown in these figures, with spectra taken from three to five samples for each optical fluence. The results shown here are used to define the mean and standard deviation for both the peak heights and the ratios of peak heights at each optical fluence.
